# Laying the Foundations for Co‐Design: A Micro‐Community Engagement Approach to Patient and Public Involvement in Rural Health Research

**DOI:** 10.1111/hex.70816

**Published:** 2026-08-16

**Authors:** Louise K. Wiles, Aaron Davis, Ian Gwilt, Hayley B. Leake, Ashley R. Grant, Katrina Martin, Kate Gunn, Sarah B. Wallwork, David Wearing, Emma Young, Julie Di Rito, Ian McKay, Jared Simes, G. Lorimer Moseley, EQUiPP Group

**Affiliations:** ^1^ IIMPACT in Health, Allied Health and Human Performance, College of Health Adelaide University, Kaurna Country Adelaide South Australia Australia; ^2^ College of Creative Arts, Design and Humanities Adelaide University, Kaurna Country Adelaide South Australia Australia; ^3^ Western Victoria Primary Health Network Geelong Victoria Australia; ^4^ Office of the Commissioner for Public Sector Employment Adelaide South Australia Australia; ^5^ Country SA Primary Health Network Nuriootpa South Australia Australia; ^6^ Office of the Provost and DVC Mount Gambier Adelaide University, Boandik Country Mount Gambier South Australia Australia; ^7^ Lawson Risk Adelaide South Australia Australia

**Keywords:** chronic pain, co‐design, community engagement, community networks, patient and public involvement (PPI), rural health

## Abstract

**Background:**

Rural communities face barriers to accessing, sharing and trusting health information, which can limit the relevance and impact of health education initiatives. Early engagement to understand local communication networks is critical for designing effective, community‐grounded co‐design processes.

**Objective:**

To explore, with community members, how health information is accessed, shared and trusted within rural towns to inform a subsequent co‐design process.

**Design:**

Formative participatory engagement study using qualitative methods, including participatory mapping, semi‐structured interviews and field notes conducted within existing community awareness events.

**Setting:**

Seven rural towns in South Australia (SA) and Victoria (VIC), across Modified Monash Model (MMM) categories: 3 (Horsham, Mount Gambier), 4 (Ararat, Hamilton, Naracoorte and Stawell) and 5 (Millicent).

**Participants:**

Adults who attended the events, local healthcare professionals (HCPs) and/or community interest‐holders.

**Intervention:**

Patient and public involvement and engagement (PPIE), hereafter referred to as community engagement, comprised participatory mapping activities, informal ‘stall‐side’ discussions and semi‐structured interviews conducted during community awareness events. The maps, interview transcripts and field notes generated through these activities constituted the study data.

**Main Outcome Measures:**

Engagement activities generated three forms of data: participant‐generated map locations, interview audio‐recordings with consenting participants and researcher field notes.

**Results:**

A total of 205 members of the public and interest‐holders and 59 HCPs participated; 300 community connection and information‐seeking points were identified through 282 stickers placed on town maps. Interviews were completed by 35 participants. Formal information‐seeking and communication networks varied across communities but included sporting clubs; HCPs and health services; council amenities and venues, and community clubs and associations. Informal networks included special interest groups, eateries, hotels/pubs and community events. Interview data provided additional context about how these networks operate and which populations they most effectively reach.

**Discussion and Conclusions:**

Future co‐design and public messaging efforts should reflect both shared regional pathways and locally specific communication networks, incorporating region‐wide and community‐specific engagement strategies to support meaningful community participation.

## 1 Introduction

Improving public understanding of pain and its management is increasingly recognised as a public health priority [1]. Misconceptions about pain can influence care‐seeking, treatment choices and outcomes, and contribute to low‐value care [1, 2, 3, 4]. Education is recommended as a first‐line treatment for chronic pain [5, 6], and previous work has identified both general and condition‐specific learning goals for effective pain education [7, 8, 9]. However, access to such education remains limited in rural areas, where geographic isolation, workforce shortages and variability in service availability contribute to persistent inequities in health literacy and health outcomes [10, 11]. A recent systematic review found that up to half of public health messaging programs risk worsening inequities when they overlook social and economic factors or fail to reflect community contexts and needs [12]. Programs that are designed and developed with communities, recognising population heterogeneity, are therefore recommended [11, 13].

Early engagement with partner organisations and regional interest‐holders highlighted a commonly held assumption that may undermine public health initiatives: that communities within rural regions are broadly similar and can be reached using uniform strategies [14, 15]. However, this assumption overlooks important differences in how communities communicate, share information and engage with local services. Public messaging approaches that combine locally tailored strategies with transferable methods may therefore be most effective in optimising reach and impact.

Meaningful patient and public involvement in health initiatives requires more than inviting participation; it requires understanding how people already connect, communicate and seek information within their communities. Although co‐design and participatory approaches are increasingly advocated in health research and service development, evidence suggests that their implementation is variable, with limited reporting of how engagement is embedded within local systems and how participants are initially identified and reached [16, 17, 18]. In particular, relatively little attention has been given to the pre–co‐design phase, where the social and communication infrastructures that shape participation are identified and mapped. Understanding these local pathways may help ensure that subsequent co‐design activities occur within networks, locations and relationships that are meaningful to participants.

This study therefore sought to explore community perspectives on how rural populations can best be reached and engaged in a future co‐design process. By identifying locally legitimate pathways for participation prior to intervention development, this formative work aimed to strengthen the relevance and feasibility of subsequent co‐design activities.

## 2 Objective

To identify, using a precision participatory approach, where, when and how people in seven rural towns access and exchange credible health‐related information.

Engagement was undertaken at a micro‐community (town‐by‐town) level. To our knowledge, this represents the first reported use of such an approach as a precursor to co‐designing a health education intervention with rural and regional communities.

## 3 Methods

Ethics approval was obtained from the Adelaide University (formerly University of South Australia) Human Research Ethics Committee (Protocol: 206208). Reporting of qualitative components was informed by the SRQR (Standards for Reporting Qualitative Research) [19].

### 3.1 Patient and Public Involvement

Community members were involved in shaping the engagement approach through prior informal consultations with local organisations, clinicians and consumer advisory groups. These discussions informed the design of the participatory mapping activity and interview prompts. Findings from this study will inform the subsequent co‐design phase of the EQUiPP project. This formative engagement phase was designed to ensure that subsequent co‐design activities are conducted through networks, locations and communication channels that communities themselves identify as accessible and trusted.

### 3.2 Design and Setting

This was a formative participatory engagement study using qualitative methods, undertaken in seven priority towns, identified through regional needs assessments [14, 15] across Modified Monash (MMM) categories [20]: 3 (Horsham, Mount Gambier), 4 (Ararat, Hamilton, Naracoorte and Stawell) and 5 (Millicent).

Data were collected during nine community engagement events organised by *Pain Revolution* [21], as part of their *2024 Rural Outreach Tour* [22]. These *Pain Revolution* events focused on pain education and evidence‐based strategies for chronic pain prevention and recovery.

Two events (Mount Gambier, Horsham) were full‐day workshops, targeting knowledge and skills development for healthcare professionals (HCPs). The remaining seven events were 90‐min community sessions combining information presentations with interactive activities. Each event also included ‘marketplace stalls’, where attendees could engage with *Pain Revolution* presenters and members of the research team, access free printed information resources on chronic pain and its management, and interact with educational displays designed to communicate key principles of pain science and evidence‐based chronic pain care. Data collection occurred within these marketplace areas before, during and after the *Pain Revolution 1* events.

### 3.3 Participants

Attendees at *Pain Revolution's 2024 Rural Outreach Tour* community events (14–21 September 2024) were eligible to participate. The study sample therefore comprised adults (aged 18 years and over) who lived in or near one of the participating communities and who either:

i. had lived experience of chronic pain or an interest in the topic, or
ii. represented organisations with an interest in chronic pain (e.g., local employers, sporting clubs and government agencies), or
iii. were HCPs.

To support informed participation, study information sheets outlining objectives were made available online and in print prior to each event. No individual‐level demographic data (e.g., age, gender, occupation and ethnicity) were collected as part of this study, because we wanted to minimise burden and support participation in a naturalistic community engagement setting.

### 3.4 Data Collection

Two complementary data collection activities were conducted at the marketplace stall:

i. *Participatory mapping*, where participants placed custom stickers on large‐format town posters (A0; 841 × 1189 mm) displayed on easels. These posters represented 14 towns: the seven target sites and seven surrounding districts identified by partner organisations as important community hubs [14, 15].
  Participants were asked to identify locations where people access or exchange health information.
ii. *Semi‐structured interviews* were conducted using a flexible interview guide (Table 1). Verbal consent was obtained prior to participation and reconfirmed at the outset of each interaction. In practice, interviews were delivered in a conversational manner to accommodate the informal, time‐limited and embedded nature of the community event setting. All attendees were invited to participate, with interactions ranging from brief, informal conversations to more in‐depth discussions. Researcher field notes captured detailed interview discussions and brief conversational exchanges conducted within the same consented interaction.

**Table 1:** Interview schedule.

Screening questions:
Are you aged 18 years or over?
Do you live or work in one of the following seven towns, or their surrounding districts:
Mount Gambier, Naracoorte, Millicent, Hamilton, Ararat, Stawell or Horsham?
Interview questions:
1. **Why did you come here today?** *Prompts: Are you a healthcare professional? Do you have pain yourself? Do you know/care for/support someone who has pain?*
2. **What did you get from today's/tonight's session?** *Prompts: What was the most important message that you gained from the session? Why/in what way? What might you do [with that message/information] from here?*
3. **Where do people in the town connect to share general information?** *Prompts: How do you usually find out about what's going on in town? For example, football/netball club, Country Women's Association [CWA], Rotary Clubs, library, local hotel/pub, school, work*.
4. **Who are the key people in the town that know everything that is going on?** *Prompts: Mayor, Member of Parliament, local organisations/employers, local school Principal ± sports coaches, farmers, philanthropic organisation staff*.
5. **Where do people in the town go to get health information?** *Prompts: General practitioner [GP], pharmacist, local newspaper or television/radio news service, social media, council newsletters, sports club, local community events*.
6. **Who are the most trusted people or organisations in the community for getting health advice?** *Prompts: General practitioner [GP], pharmacist, local newspaper or television/radio news service, social media, council newsletters, sports club, other local community organisations*.
7. **How could we best reach different groups in the town?** *Prompts: Young people, young families, middle‐aged people, early retirees, later retirees, older people; how to advertise the EQUiPP project, days/times/seasons to recruit, modes/methods of involving the community*.

Interview data were collected using two approaches depending on participant consent: where participants agreed, interviews were audio‐recorded and transcribed verbatim; where participants did not consent to audio‐recording, data were captured through contemporaneous field notes completed by the researcher during and immediately following the interaction. Both audio‐recorded transcripts and field notes were included in the analysis.

The interviewer (L.K.W.), a physiotherapist and health services researcher, had prior experience in primary care and hospital settings, including chronic pain care, and had engaged with local interest‐holder organisations during earlier stages of project planning. Reflexive field notes were maintained throughout data collection to document contextual observations and researcher reflections, enabling ongoing consideration of how the researcher's background and interactions with participants may have influenced data collection and interpretation.

The mapping activity and interviews were intentionally designed as complementary. Participatory mapping identified locally relevant information networks, while interviews provided contextual explanation of how and why these networks functioned within each community. Consequently, interviews were not intended to achieve thematic saturation independently but to elaborate and contextualise findings from the mapping activity.

### 3.5 Analysis

A six‐stage collaborative thematic analysis was conducted for the town poster data:

1. planning,
2. open and axial coding,
3. preliminary codebook development,
4. pilot testing,
5. final coding and
6. codebook review to finalise themes on formal and informal networks across target towns.

Coding was completed by one researcher (Researcher 1) and cross‐checked by a second researcher (Researcher 2). Descriptive statistics were used to summarise theme frequencies. Interview data were analysed thematically and compared with the poster‐derived codebook to support, refute, expand or contextualise emerging themes. Analytical decisions were discussed between researchers (L.K.W., A.D., and I.G.) to strengthen interpretive rigour. For descriptive purposes, identified networks were grouped into ‘formal’ and ‘informal’ categories. Formal networks comprised organisations or institutions with identifiable governance structures and/or established communication pathways, whereas informal networks comprised settings or groups where information exchange occurred more organically through shared interests, social interaction or everyday community activities.

## 4 Results

Findings are presented in three parts: cross‐regional patterns in information‐seeking networks, variation in network distribution across towns and community‐identified health priorities.

A total of 264 individuals attended the events, including 205 members of the public and interest‐holders, and 59 local healthcare providers (HCPs). Attendees placed 282 custom stickers endorsing 300 community connection and information‐seeking points across 14 town‐specific posters. Thirty‐five participants (22 public/interest‐holders [63%], 13 HCPs [37%]) took part in interviews, with 6 (17%) consenting to audio‐recordings (mean duration: 18 min 12 s; range: 4:56–45:43).

Interviewees proposed 170 strategies for community engagement; all strategies were consistent with the themes identified in the poster data, and no contradictory perspectives were observed. Four additional ‘important communities’ were named: Kalangadoo (SA), and Dimboola, Branxholme and Halls Gap (VIC). Independent verification of the town poster and interview codebooks confirmed alignment across themes.

### 4.1 Cross‐Regional Patterns in Information‐Seeking Networks

Formal networks for information‐seeking and communication demonstrated clear commonalities across communities, while still varying in their relative prominence between towns (Table 2). These included: sporting clubs (e.g., football, netball; *n* = 37, 12.3% of all suggestions), local HCPs and services (e.g., hospital, general practitioners, physiotherapists, pharmacists and psychologists; *n* = 37, 12.3%), local council amenities/venues (e.g., libraries, community centres/halls/noticeboards and council chambers; *n* = 35, 11.7%) and community clubs/groups and associations (e.g., Lions, Rotary, Probus and Returned and Services League [RSL], *n* = 33, 11%). Informal networks included: special interest groups (e.g., historical societies, quilting/arts/crafts, garden/agricultural clubs, Men's Sheds and ‘Mums and Bubs’; *n* = 27, 9%), local eateries and hotels/pubs (*n* = 17, 5.7%) and events (e.g., specific annual shows, field days, and festivals and farmers' markets; *n* = 16, 5.3%).

**Table 2:** Formal and informal networks operating in each of the towns. Frequency of formal and informal networks operating in each town:

1–2	3–4	5–6	7–8

Town	Formal networks									Informal networks									Total
	Government/local government	Local council amenities/venues	Local council services	Employers/workplaces	Schools	Community clubs/groups/associations	Sporting clubs	Local healthcare professionals/services	Local media	Social media pages	Special interest groups	Local/general store, other retail	Local eateries/hotels (pubs)	Exercise venues/services	Other social venues	Local parks/nature and conservation areas	Events	Other	
Mount Gambier	5	3	2	1	1	3	3	7	3	1	3		2	2	4	1	2	3	46
Millicent		5	1		1		2	3	1		1		3						17
Naracoorte	1	4	1		1	5	1	4		1	4			2		1	1		26
*Beachport*	1	2		1	2			3	1	1	1		2						14
*Lucindale*							1										1		2
*Coonawarra*													1						1
*Penola*	1	1	1			2	2				1			1			3		12
*Padthaway*												1							1
*Bordertown*		1			1	2	1	2	1										8
Kalangadoo*							1			1			1						3
Hamilton	1	6	2		1	2	7	4	1		3	2	1	2	2		2		36
Ararat		8	2	1		6	2	4	1		4	1	1	4	2		2	1	39
Stawell		3	3			5	6	5	1	2	7	1	1	3		3	2		41
Horsham		1	1	1	1	4	8	3		1	1		2	1	4		2		30
*Nhill*	2	1	1		1	1	3	1			2	1	3		1		1	1	19
Dimboola*															1			1	2
Branxholme*																			
Halls Gap*			1			1		1											3
Total	11	35	15	3	8	33	37	37	9	6	27	6	17	15	14	5	16	6	300

*Note:* A total of 282 individual locations were identified; however, some had dual purposes (e.g., library as a venue, and a service) which led to a higher total number of networks (*n* = 300). The additional context was captured in the interview data, highlighting the importance of multi‐method data collection approaches. Target towns, *Surrounding districts identified as ‘important communities’ by our partner organisations*. *Additional towns identified by participants as being ‘important communities’.

Qualitative interview data provided further detail and context about the networks, including how they operated and which populations they most effectively reached (Table 3). Participants frequently emphasised the role of local ‘everyday’ infrastructure, such as general stores, pubs and community groups, in circulating informal but credible health‐related information. For example, one participant noted that ‘the general store owner seems to know everything going on ‐ we go to the shops to get all the local news’, highlighting the embedded social role of retail spaces in rural information sharing. Similarly, community clubs and sporting organisations were described as key engagement points, with one participant stating that ‘footy clubs are really good at getting behind a cause’, reflecting their perceived reach and influence. Health and community services also featured strongly, with participants identifying Aboriginal Community Controlled Health Services, pharmacists and local councils as trusted sources of information and support. These accounts reinforced the mapping findings while also clarifying the social mechanisms underpinning trust, including familiarity, routine attendance and interpersonal connection.

**Table 3:** Qualitative interview data examples that provided additional detail about the formal and informal networks.

Networks	Categorisation	Evidentiary interview quotes/field notes	Interview	Participant	Location
Formal networks	Government/local government	‘Centrelink, maybe? I imagine there would be people there who could benefit from this sort of information.’	3	HCP	Mount Gambier
		‘There's a Town Information Centre that quite a few people go through.’			
	Local council amenities + venues	‘The Library have free newspapers and books of course, it's a nice space. They run talks and sessions too.’	13	Community	Hamilton
		‘The Library here runs information sessions for awareness weeks or months, and there are other council initiatives.’	29	HCP	Horsham
	Local council services	‘They host a Wellbeing Program at the Town Hall ‐ it's for being active and socialising.’	9	Community	Naracoorte
	Employers/workplaces	‘Lunchtime learning sessions at workplaces could be an option?’	28	Community	Horsham
	Schools	‘School newsletters are underutilised but good for getting information to families, not necessarily for kids' information but about general activities and goings on.’	10	HCP	Naracoorte
		‘Schools are not a good option around here. Most children go to schools in other larger towns. There are only 100 homes in Kalangadoo.’	2	Community	Mount Gambier
	Community clubs/groups/associations	‘In Beachport, Lions Club is key.’	8	Community	Naracoorte
	Sporting clubs	‘Footy Clubs are really good at getting behind a cause.’	27	Community	Horsham
		‘EQUIPP could advertise in the Footy Budget. Everyone who goes to the game buys oneone.’	10	HCP	Naracoorte
	Local healthcare services/professionals	‘The Aboriginal Cooperative. It's a community health centre ‐ it has medical and a gym.’	26	HCP	Horsham
		‘There's the pharmacist too, especially when you can't get in to see your GP, plus it's free.’	2	Community	Mount Gambier
	Local media	‘There are the local newspapers. There's Border Watch ‐ that's for the region. There's the South Eastern Times in Millicent, the Penola Pennant, and Naracoorte News. There's the SE Voice as well but I think that's only online now.’	2	Community	Mount Gambier
Informal networks	Social media pages	‘The Beachport Community Group has a Facebook page. Everyone looks at that.’	5	Community	Millicent
	Special interest groups	‘Mums' Groups often meet in a pub or café restaurant ‐ you could probably access them through Maternal and Child Health Services.’	28	Community	Horsham
		‘There's the Theatre Group, Brass Band, Men's Sheds – women can go too.’	18	Community	Ararat
	Local store/general store/other retail	‘The general store owner seems to know everything going on ‐ we go to the shops to get all the local news!’	2	Community	Mount Gambier
		‘Op shops ‐ they're in touch with lots of different people. They also offer other support for finance, needing food ‐ they're connected to Food Bank.’	4	Community	Millicent
	Local eateries and hotels/pubs	‘There are a few pubs that host quiz nights ‐ The Federal and Woolstore Brewery.’	3	HCP	Mount Gambier
	Exercise venues/services	‘The gym runs exercise sessions for older people ‐ there's 10‐14 people in each group. It's fitness but we socialise too, chat about things…’	9	Community	Naracoorte
	Other social venues	‘There's ten pin bowling; Inflatable World, Laser Tag and indoor netball at Dimboola ‐ it's a pretty popular town around 25 minutes away. People go there on weekends.’	30	HCP	Horsham
	Local parks/nature + conservation areas	‘Cato Park is good. Lots of people walk around the lake, there's some fishing there as well.’	22	HCP	Stawell
	Events	‘You could have an information booth at the Farmers' Markets. Most towns have Farmers' Markets and ours gets a big cross‐section of people who go.’	3	HCP	Mount Gambier
	Other	‘The guy who runs Limestone Coast Community News ‐ he's got a lot of followers and is generally a key figure in the community.’	4	Community	Millicent

### 4.2 Town‐Level Variation in Network Distribution

While common types of networks were observed across all sites, the relative distribution and frequency of these networks varied across towns (Table 2). Larger regional centres demonstrated a broader range and higher frequency of identified network types, whereas smaller rural towns showed more limited but highly locally embedded networks.

### 4.3 Community Health Priorities

When prompted to identify the most pressing health concern in their community, participants selected from predefined categories including mental health, influenza/COVID‐19, stroke/heart disease, chronic pain and obesity/diabetes. Mental health was the most prioritised category across sites based on participatory mapping responses, and this pattern was also reflected in interview data.

## 5 Discussion

This study engaged rural communities at a micro‐community level to understand how people locally access, share and trust health information, as a foundation for meaningful patient and public involvement in subsequent co‐design. Our findings suggest that effective patient and public involvement depends not only on inviting participation but also on recognising the local networks through which participation is most likely to occur.

Participatory and co‐design approaches are increasingly used in health research [23], yet less is known about how engagement strategies can be developed during the preparatory stages of co‐design. Our approach combined participatory mapping with semi‐structured interviews conducted flexibly within the existing community events. Together, these methods provided complementary insights into locally trusted networks, key gathering spaces and communication pathways within and across rural towns, identifying how information circulates and which sources are trusted among community members.

Beyond informing the EQUiPP project, this study illustrates a practical approach to early‐stage community engagement in health research. Mapping information networks prior to intervention development may help ensure that subsequent co‐design activities occur through channels that communities themselves recognise as legitimate and accessible. This preparatory phase therefore represents an important, but often underreported, step in meaningful patient and public involvement.

Three key insights emerged from this work. First, participants emphasised the central role of grassroots community networks in sharing information. Sporting clubs, healthcare providers, council services and community groups were consistently described as trusted social infrastructure within rural towns. Notably, participants rarely identified commercial or mass media channels, with only one community radio station mentioned. This aligns with previous rural health research highlighting the importance of place‐based relationships, local context and trusted community networks in supporting meaningful engagement and information exchange [24, 25].

Second, although certain types of networks were common across towns, the specific locations and social spaces through which information circulated were largely community‐specific. This finding suggests that effective engagement strategies may need to operate at both regional and town‐level scales, rather than relying solely on conventional region‐wide approaches. This reinforces broader literature on rural health engagement and co‐design, which highlights the importance of responsiveness to local context and social infrastructure rather than assuming homogeneity across regions [26].

Third, when asked about the most pressing health concern within their communities, participants most frequently identified mental health. This is consistent with broader Australian rural health evidence identifying mental health as a persistent and significant community concern [27, 28]. Given the well‐established relationship between chronic pain and psychological wellbeing [29], these findings highlight the potential value of framing pain education initiatives in ways that acknowledge the intersection between pain and mental health. However, given ongoing stigma and sensitivities surrounding mental health in some rural communities [30], such approaches should be developed carefully and in partnership with local communities.

Taken together, these findings extend existing co‐design and patient and public involvement literature by demonstrating the value of an explicit pre‐co‐design mapping phase that identifies trusted communication pathways before intervention development. While co‐design approaches emphasise participation and collaboration [23], less attention has been given to how the engagement infrastructures that enable meaningful participation can be empirically identified, understood and tailored to local contexts. This study therefore contributes a practical methodological step between initial interest‐holder engagement and formal co‐design, strengthening the early ‘architecture’ of participatory health research in rural settings.

Our findings extend this work by demonstrating a micro‐community, place‐based approach that identifies both shared regional patterns and highly localised communication infrastructures across rural towns. This aligns with emerging evidence that rural health co‐design is strengthened when it is responsive to local context, relationships and systems rather than relying on uniform or region‐wide engagement strategies [26]. In doing so, it contributes a novel methodological step between community consultation and formal co‐design, an area that remains underdeveloped in current participatory health research literature.

This study has limitations. Although *Pain Revolution* events were open to all ages, participants under 18 years were excluded from this study. Individual‐level demographic data (e.g., age, gender, occupation and ethnicity) were not collected, so as to minimise participant burden and support engagement in a naturalistic community setting. We acknowledge that this limits our ability to fully characterise participants' social contexts or understand which population groups were represented within the engagement activities [31, 32].

While we did not expect *Pain Revolution* events to attract marginalised groups, some insights emerged—for example, participants made suggestions that extrapolated beyond their immediate experience (refer Table 3; e.g., Centrelink, pubs). Data collection activities were feasible to implement and appeared acceptable to participants (i.e., attendees revisited town posters and/or engaged in interviews after the information sessions, and several participants expressed appreciation for researchers ‘coming to them’). Most interviewees declined audio‐recording, which may reflect the informal and conversational nature of the marketplace engagement setting. Although this limited the number of verbatim transcripts available, contemporaneous field notes enabled all interviews to contribute to the analysis. Because participants' reasons for declining audio‐recording were not systematically explored, a formal evaluation of the engagement approach could have identified opportunities to refine future data collection methods while preserving participant acceptability. In grouping networks as ‘formal’ and ‘informal’, we recognise that some settings could reasonably fall into either category. These labels were therefore used as a pragmatic analytical framework to describe the predominant ways participants characterised information exchange within their communities. Finally, we did not pre‐register our protocol, a recommended practice in pain research [33], which would have improved transparency. However, this is less common in formative research.

Study strengths included an approach shaped by informal conversations with community members, local industry and organisations (e.g., Primary Health Networks, employers, councils, sports and community groups), independent feedback from clinician and consumer advisory groups [34], and purposive sampling. Conducting engagement activities within existing community events enabled participation within familiar and trusted environments, which may have supported more naturalistic discussions. As the intent was exploratory and formative, qualitative data were analysed inductively rather than within a predefined theoretical framework. Given the modest sample size and the exploratory, formative nature of this work, the data were interpreted as naturalistic accounts, with emphasis on nuance and contextual insight rather than generalisable patterns. This participant‐led approach allowed us to view data extremities as potential ‘lead use cases’ rather than outliers [35], helping build a rich picture of each town's sociocultural context for the next phase(s) of the [anonymised project].

In conclusion, we developed and applied a precision participatory approach to engage rural communities at a micro‐community (town‐by‐town) level. This approach generated both region‐level and town‐specific insights to inform the next phase of the project: community co‐design. Findings highlight the importance of working through locally trusted networks and venues, and of tailoring engagement strategies to the social infrastructure of individual rural communities. Reporting this preparatory engagement work may help address the gap between calls for co‐design and the practical realities of building locally grounded and socially legitimate participation in health research.

## Author Contributions

**Louise K. Wiles:** funding acquisition, data curation, project administration, formal analysis, methodology, writing – original draft, conceptualization, investigation. **Aaron Davis:** funding acquisition, conceptualization, investigation, writing – review and editing, methodology, validation, formal analysis. **Ian Gwilt:** conceptualization, investigation, methodology, validation, writing – review and editing, formal analysis. **Hayley B. Leake:** funding acquisition, investigation, methodology, writing – review and editing. **Ashley R. Grant:** investigation, methodology, writing – review and editing. **Katrina Martin:** funding acquisition, investigation, writing – review and editing. **Kate Gunn:** funding acquisition, investigation, writing – review and editing. **Sarah B. Wallwork:** investigation, writing – review and editing. **David Wearing:** writing – review and editing, investigation. **Emma Young:** investigation, writing – review and editing. **Julie Di Rito:** investigation, writing – review and editing. **Ian McKay:** investigation, writing – review and editing. **Jared Simes:** investigation, writing – review and editing. **G. Lorimer Moseley:** funding acquisition, conceptualization, investigation, writing – review and editing, methodology, supervision.

## EQUiPP Group

The following listed members of the EQUiPP Group are Chief and Associate Investigators on the wider project and have contributed to the overall EQUiPP protocol development: Elizabeth E. Roughead, John W. Toumbourou, Adrian C. Traeger, Peter D. Hibbert, Anne L. J. Burke, Katherine Graham, Virginia Mumford, K. Jane Chalmers, Adrian Esterman, Christopher M. Williams, Sue Nichols, Florence Gabriel, Felicity A. Braithwaite, Emma L. Karran, Alison Kennedy and Alex Collie.

## Conflicts of Interest

L.K.W. has received support to attend conferences and speaker fees for lectures on appropriateness of care and consumer engagement. H.B.L. and S.B.W. have received speaker fees for lectures on pain and rehabilitation. G.L.M. has received support from: Reality Health, CORA UK, Institutes of Health California, AIA Australia and Workers' Compensation Boards and professional sporting organisations in Australia, Europe and South and North America. Professional and scientific bodies have reimbursed him for travel costs related to presentation of research on pain and pain education at scientific conferences/symposia. He has received speaker fees for lectures on pain, pain education and rehabilitation. He receives royalties for books on pain and pain education. He founded and is unpaid CEO of the non‐profit Pain Revolution.

## Data Availability

The data generated and analysed during this study are not publicly available. While no individual participant identifiers were collected, the combination of location‐specific and qualitative data from small rural communities presents a risk of indirect identification. De‐identified data may be available from the corresponding author on reasonable request, subject to ethical approval and data sharing agreements.
